# Surgical Pathology and Therapeutics

**Published:** 1901-01

**Authors:** 


SURGICAL PATHOLOGY AND THERAPEUTICS, by John Collins Warren, M. D., Professor of Surgery in Harvard University; Surgeon to the Massachusetts General Hospital. Illustrated, second edition with an Appendix containing the Enumeration of the Scientific aids to Surgical Diagnosis, together with a series of sections on Regional Bacteriology; 873 pages; price, $5.00; Philadelphia: W. B. Saunders, 925 Walnut Street; 1900.

A knowledge of Surgical Pathology is the basis of good surgery, and is necessary to a practical success. The time was when a scientific surgical education was regarded as more ornamental than useful, but it is now demanded of every student by every school. The surgeon who does not possess a working knowledge of pathology and bacteriology has a limited field.

The author has successfully set forth in the above work the associate pathological conditions with the symptoms and treatment of surgical diseases and in a style most attractive and convincing. There is perhaps no book published this year that gives a more thorough, up-to-date discussion of surgical pathology and therapeutics than Warren's, and it is endorsed most cordially.

